# Supplementary material for: Are carbon emissions trading and green financial instruments synergistic? -Comprehensive quantitative research based on content analysis
Source: PLoS One. 2024 Mar 7;19(3):e0298601. doi: 10.1371/journal.pone.0298601 (PMC10919601; doi:10.1371/journal.pone.0298601)
Supplement: S1 Data — (ZIP) [file pone.0298601.s001.zip › Data/地级市Z_F工作报告.zip!/┴╔─■/╕o╦│╩╨/└┤╫╘╣1⁄2╓┌║┼í╛┬φ┐╦╩2╛▌═°í┐_2008.docx]

各位代表：

　　现在，我代表市十三届人民政府向大会报告工作，请予审议，并请政协委员和其他列席人员提出意见。

　　一、2007年暨本届政府工作回顾

　　2007年是本届政府任期的最后一年。一年来，在市委的正确领导下，在市人大和市政协的监督支持下，我们紧紧抓住振兴东北和推进沈抚同城化的重大机遇，团结带领全市人民，致力科学发展，促进社会和谐，圆满完成了市人大十三届五次会议确定的各项目标任务。

　　国民经济持续快速增长。预计实现地区生产总值547亿元、增长16.1%，其中第一产业增长10%，第二产业增长16.9%，第三产业增长16.1%；高新技术产业增加值70亿元，增长28%；固定资产投资281亿元，增长40%；社会消费品零售总额234亿元，增长15.6%；地方财政一般预算收入36.7亿元、支出74.5亿元，分别增长33.3%和22.4%；城市居民人均可支配收入和农民人均纯收入达到10800元和4800元，分别增长16.1%和15.4%；万元生产总值能耗降低4.6%。

　　项目建设成效显著。大力开展“项目年”活动，全年引进域外项目882个，到位资金156.4亿元、增长28.4%；其中投资超5000万元项目108个、超亿元项目60个，辽宁重型电机制造等一批大项目开工。引进境外项目42个，实际利用外资6890万美元、增长30.5%，创历史最高水平。年初确定的120个重点项目开工110个，完成投资222亿元，开工率高于上年3个百分点；其中工业项目71个，完成投资125亿元。

　　结构调整积极推进。地方工业特别是县域经济发展加快，新增规模企业172户；“两区四园”达到项目摆放标准，项目开工111个、总投资67亿元。有机化等30户国企完成改制，新增民营规模工业企业120户、增长22.5%，新钢铁和罕王集团跻身全国民企500强；民营企业实现增加值267亿元、税金30亿元，分别占全市总量的49%和55%。新批外贸自营权企业30家，外贸出口6.5亿美元、增长24%，平天蜡制品被评为省出口名牌产品。中兴时代广场、金汇广场等大型商业中心建设进展顺利，新增商业网点2000个、标准农家店240家。红河峡谷漂流晋升国家4A级旅游景区，皇家极地海洋世界二期、平顶山惨案纪念馆改造等一批旅游项目竣工，新增农家乐80家，满族风情国际旅游节圆满成功，实现旅游总收入57.7亿元、增长23%。

　　沈抚同城化全面启动。沈抚两市成功举办高峰论坛，签署了《加快推进沈抚同城化协议》，初步确定两市共同开发区域和产业发展方向，规划了总投资215亿元的8大重点项目，并得到开发银行149.2亿元信用合作额度支持，其中首批10.5亿元资金已经到位。沈抚连接带、产业园区、浑河生态环境等45个基础设施项目建设已经起步，并完成投资13亿元。总投资142.9亿元的浑河污染源治理工程全面启动，污水处理厂、中水回用等项目完成投资2.7亿元。三宝屯沈抚高速公路收费站已经取消，沈抚大道顺利开通，沈抚客运实现公交化。

　　新农村建设进展较快。战胜严重自然灾害，粮食产量达到54.8万吨。新增规模以上龙头企业12户，辐射带动农民12.8万户，形成特色专业乡镇7个、产业专业村115个，建立并规范农产品标准化生产基地116万亩、增长84%，塔峪、海浪等一批设施农业基地基本建成；畜牧业“清洁生产示范市”建设效果明显，新增标准化畜牧生产小区15个，清原马鹿茸和抚顺哈什蚂通过国家地理标识认证。国家级“现代林业示范市”建设实现良好开端，集体林权改革完成869万亩，确权到户率达96%以上；“水果采摘月”活动深受城乡群众欢迎。新建“一池三改”4510个、秸秆气化站10处。打击非法采砂和破坏林地专项行动取得阶段性成果，罚款及新增税费2260万元；治理水土流失面积12.1万亩，12万人饮水难得到解决；夺得全省农建“大禹杯”。大中型水库后期移民扶持首批资金和项目已经到位，参加新农村帮建的108个行政村生产生活条件得到改善，2.3万人实现稳定脱贫。

　　城乡环境建设力度加大。高山路西段、葛布通道、城市西出口、劳动公园改造等一批标志性城建项目建成使用；改造城市道路110条59.6公里76.6万平方米、“摸黑路”32条23公里；绿化重点街路29条，植树造林134.4万株，新增绿地78.7公顷。高速公路南杂木至草市段开工建设，南环公路、彰桓线清原至新宾段竣工通车；新建农村黑色路面300公里，新增通油路村58个。绿化硬化小区43个103万平方米，整治浑河干支流18公里；对浑河城市段中部两岸进行了亮化，浑河两岸的夜景更加迷人。“抚顺行动”环保工程全面启动,城市空气质量达到优良标准天数首次突破300天，浑河和大伙房水库水质分别达到四类和保持二类标准，减排幅度位居全省第二。实现土地出让收入18.5亿元，增长186%。

　　民生状况持续改善。坚持棚改一号民心工程不动摇，拆迁棚户区97.75万平方米，建设安置用房136万平方米，建设规模和回迁速度全省第一，又有2.6万户、7.9万人喜迁新楼。全市实名制就业14.2万人、普惠制培训5.3万人，零就业家庭全部实现至少一人就业，其中90%实现当年双就业，棚改回迁居民就业率达到92%。强力推进养老保险扩面征缴，新增养老保险参保企业611户、2.9万人，22.8万人全部按时足额领取了调增的养老金。加强城乡低保动态管理，确保了困难人群应保尽保。强化信访工作责任制，信访总量下降5.1%。打击刑事犯罪综合绩效考评全省第一，社会治安秩序明显好转，群众安全感明显增强。依法关闭煤矿、非煤矿山和小炼油厂26户，生产安全事故得到有效控制。承诺为市民办的20件实事全部完成。

　　社会事业全面发展。建成国家级重点实验室2个、企业技术中心2个，省部级重点实验室8个、工程中心6个，抚顺精细化工应用技术研究院挂牌成立，抚顺获批成为全国首家国家级精细化工产业化基地。这对于推进百万吨乙烯后续产业发展、加快资源深加工意义十分重大。新一轮“普九”通过省验收，提前一年实现规划目标；全部消灭农村学校新增D级危房和城市学校旱厕，新建农村九年一贯制学校6所，维修改造棚户区学校15所；新建改建公共实训基地9个，职业院校办学水平明显提高；加大对贫困生资助力度，因贫困而失学的现象基本杜绝。农民参加新型合作医疗62.9万人，占农业人口的86.8%；城市社区卫生服务覆盖率100%。中央心连心艺术团首次来抚演出，中央人民广播电台直播中国走进抚顺，话剧《那座山村那条路》获省艺术节金奖，煤精雕刻、满族剪纸、清原民间故事被列入省非物质文化遗产名录。成功承办红河峡谷国际漂流等赛事，新建全民健身场所105处。人口出生率和自然增长率分别控制在5.26‰和0.19‰以内。

　　政府自身建设进一步加强。自觉接受人大法律监督和政协民主监督，坚持重大决策向人大常委会报告、向政协通报制度； 806件市人大代表建议和政协提案全部办结，满意率均达99％以上。市长公开电话转办件解决率达98%。认真贯彻《全面推进依法行政实施纲要》，清理废止政府规章86件，公共行政服务中心的服务水平进一步提高。市属399家事业单位完成改革298家。深入开展“双评”活动，开办“行风政风热线”和“抚顺民心网”，加大纠风专项治理和绩效考核力度，地区发展软环境进一步改善。工商、质监、税务、国家安全、海关、检验检疫、食品药品监管、气象等各项事业都取得了新成绩。

　　各位代表，2007年各项任务的全面完成，为本届政府画上了圆满句号。五年来，经过全市上下的团结奋斗和务实苦干，各行各业、各个领域都发生了全面、深刻的历史性变化。

　　这五年，是团结拼搏、务实苦干的五年。在市委的正确领导下，我们始终坚持以振兴大局为重，以百姓福祉为重，聚精会神搞建设，一心一意谋发展，殚精竭虑解难题，全力以赴促民生，全市上下形成了风正气顺、团结实干的良好氛围，广大干部群众对抚顺的认同感、归属感和自豪感明显增强。

这五年，是发展提速、实力壮大的五年。地区生产总值年均增长14.5%、比上个五年增速快4.7个百分点，财政收入年均增长22.6%、比上个五年增速快15.6个百分点，固定资产投资五年累计完成815.3亿元、比上个五年增长2.5倍。主要经济指标连创历史新高，全市经济已经步入发展的快车道。

　　这五年，是改革开放卓有成效、发展后劲显著增强的五年。基本完成具备条件国企的改制，新钢铁、抚铝、红透山矿、挖掘机等改制企业爆发力迅速释放；建立市与县区财税新体制，下放大批市属企业，强县壮区得到真正落实，各县区财政一般预算收入全部过亿。通过开展“开放年”、“项目年”活动，韩国STX、马来西亚皇家极地海洋世界等一批外资项目落户抚顺；千万吨炼油、百万吨乙烯等一批大项目开工建设；累计实际利用外资2.6亿美元，是2003年前历年总和的60%；累计完成域外引资369亿元，比上个五年总和增长2.2倍。年销售收入超亿元的民营企业由2003年的8户增加到现在的65户，民营经济占全市经济总量由三分之一提高到现在的一半。

　　这五年，是城乡面貌改观、城市价值跃升的五年。累计投入城建资金36亿元，比上个五年增长70.7%，新建和翻建天湖桥、永安桥、和平桥等跨河桥梁13座，一半以上的城区道路得到翻新改造，特别是高山路、南环公路的改造建设，以及沈吉高速公路抚顺至南杂木段的建成通车，极大提升拓展了城市的形象价值和发展空间；农村通油路村达到95.4%，城市30%的排水管网得到改造，80%的居民小区得到彻底整治。

　　这五年，是高度关注改善民生、百姓得到实惠最多的五年。累计45万人实现就业再就业，8452户零就业家庭全部实现至少一人就业；养老保险参保人员由52万增加到59.8万，连续3年增加企业退休人员养老金，并实现按时足额社会化发放，使更多的人有了稳定收入，更多的家庭得到了生活保障。城市居民人均可支配收入和农民人均纯收入分别增加4901元和1687元，城乡居民人均储蓄增加4553元。市级主要医院得到大规模改造，三县新建疾病控制中心，全市卫生医疗条件和技术水平明显提高。投资6359万元，解决居民吃水难7.7万户，特别是3万多户、9万多人彻底结束了几十年吃原水的历史，因饮用水对健康构成的损害被消除。新建住宅53万平方米，采沉区特险户全部得到搬迁安置。城市居民人均住房使用面积由2003年的14平方米增加到16.26平方米，特别是2005年实施大规模棚户区改造以来，累计改造278万平方米，新建安置用房390万平方米，7.5万户、25.3万人彻底告别棚户区，走向了新生活。棚户区改造创造了“抚顺速度”和“棚改精神”，在国内外产生了巨大影响，中央政治局5位常委来抚视察，全国160多个城市组团来抚考察学习。抚顺今天的变化有目共睹，已经具备加快振兴的坚实基础，已经步入又好又快发展的良性轨道，抚顺人开始找回久违的自信与自豪。

　　各位代表，这些成绩是在战胜严重自然灾害、克服原油大幅涨价、解决历史遗留问题的情况下取得的，实属来之不易。这是市委正确领导和人大政协监督支持的结果，是国务院、省委省政府和社会各界关心支持的结果，是全市人民齐心协力、艰苦奋斗的结果。在此，我代表市人民政府，向全市人民，向所有关心、支持和帮助抚顺经济社会发展的同志们、朋友们，表示衷心的感谢并致以崇高的敬意！

　　在充分肯定成绩的同时，我们也清醒地认识到目前存在的困难和问题：一是结构调整任务仍很艰巨，资源深加工任重道远，经济发展的速度和效益还有待提高。二是节能减排形势严峻，生态建设和保护任务繁重。三是就业再就业和社会保障压力仍然很大，部分群众生活贫困问题还未根本解决。四是机关作风、思想观念和工作效率还有差距，有些公职人员责任感和危机感不强，工作浅尝辄止、推诿扯皮和相互掣肘，甚至出现权钱交易和腐败堕落，让我们非常痛心。这些问题必须在今后的工作中切实加以解决。

　　二、未来五年奋斗目标及2008年重点工作

　　未来五年,是抚顺加快转型振兴的关键时期。国家《东北振兴规划》的全面实施和对资源型城市转型支持力度的加大，省委、省政府确定沈抚同城化战略的加速推进，为我市又好又快发展提供了难得机遇和良好环境。我市国企和集体林权改革基本完成、百万吨乙烯等一批大项目陆续建设投产、“两区四园”摆放项目空间巨大、民营经济迅猛发展、城乡基础设施日趋完善等，为我市又好又快发展注入了强大的活力和后劲。棚户区改造等民心工程的实施，一大批历史遗留问题的解决，使全市人民的凝聚力明显增强，为我市又好又快发展奠定了坚实基础。我们一定要增强责任感和使命感，解放思想，抓住机遇，同心同德，苦干实干，实现抚顺的全面振兴。这是全市人民的殷切期待，更是政府的神圣职责。

　　今后五年的总体要求是：全面贯彻党的十七大精神，以邓小平理论和“三个代表”重要思想为指导，深入落实科学发展观，紧紧抓住沈抚同城化的历史性机遇，进一步解放思想，推进改革开放和自主创新，调整产业结构，转变发展方式，提升发展质量；调整城市布局，拓展城市规模，提高城市价值和竞争力；坚持城乡统筹，加快新农村建设，促进农民增收；加强能源资源集约利用，加大环境保护和治理力度；大力发展社会事业，着力改善民生，促进社会公平，全面推进经济、政治、文化和社会建设协调发展，实现经济又好又快发展和社会更加和谐，为抚顺全面振兴而不懈努力!

　　今后五年的主要预期目标：

　　——经济实力要更大提升。地区生产总值年均增长16%以上；财政一般预算收入达到81亿元，年均增长18.5%以上；全社会消费品零售总额达到467亿元，均比2007年翻一番。科技进步贡献率达到50%，高新技术产业增加值达到190亿元，年均增长34.3%。全社会固定资产投资累计达到2000亿元，比上个五年增长1.5倍。县区经济实现跨越式发展，财政一般预算收入翻一番以上。

　　——产业结构要更趋优化。坚持工业强市不动摇，做大做强以石油化工、煤炭、冶金、装备制造等为重点的优势产业，以高新区等工业园区为主要载体的资源深加工取得突破性进展，基本建成世界知名的石油化工基地，国内有影响的新型能源原材料基地和装备制造业基地。大力发展第三产业，以旅游、商贸、房地产为重点的现代服务业成为新的支柱产业，抚顺成为全省重要的旅游城市。立足山区绿色资源，全面发展农村经济，新农村建设、现代林业建设和特色农业现代化取得明显进展，新增年销售收入超亿元的产业化龙头企业15户，农产品加工率超过60%，抚顺作为绿色特色农产品基地的地位进一步巩固。

　　——加快发展的动力机制要更加完善。国有企业和事业单位改革全面完成，集体企业改革稳步推进，企业活力和后劲显著增强；开放型经济体系基本建立，利用外资方式和结构得到创新和优化，全方位对外开放格局基本形成。五年实际利用外资5.2亿美元、域外引资1000亿元、外贸出口42亿美元，分别比上个五年增长1倍、1.8倍和1倍；民营经济不断发展壮大，年销售收入超亿元的企业增加到100户以上。

　　——城市功能和布局要更趋合理。加速“人居向浑河沿岸集中、工业向外围园区集聚”，构建“三横六纵”道路主干网络，城区东西部依托高新区、经济开发区和南环公路产业带集中摆放工业项目，形成工业“哑铃”型布局；城区沿浑河两岸地区加快“退二进三”步伐，形成人居和服务业“橄榄”型布局。推进农村城镇化，以中心镇为重点，加大基础设施建设和产业发展力度，增强其综合承载能力；加速林地、土地和人员的依法有序流动，促进城乡基本公共服务均等化，使广大农民在享受发展成果的同时，保护好青山绿水。

　　——沈抚同城化要取得更大进展。沈抚连接带核心试验区基本建成，以现代服务业和先进制造业为主的产业初具规模，成为两市极具活力的经济增长区；交通通信、商贸物流、金融服务、旅游开发、环境保护等领域的同城化基本实现。突出生态文明建设，全市森林覆盖率达到70%，位居全省前列；人均公共绿地7.5平方米，进入省级园林城市行列；城市空气质量优良天数保持310天以上，浑河干流城市段水质好于四类标准，城市建成区绿化覆盖率由现在全国排名45位前移10位。万元生产总值能耗下降15%，化学需氧量、二氧化硫、烟粉尘排放量分别削减16.3%、15%和12%以上。

　　——人民生活要更加殷实。城镇居民人均可支配收入和农民人均纯收入达到17500元和8200元以上，分别比2007年增加6700元和3400元。年均新增就业岗位2.5万个以上，比上个五年年均增加5000个，让每一个有劳动能力、有就业愿望的城镇居民都享有就业机会。以养老、医疗、低保等为重点的社会保障体系不断完善，并且覆盖广大城乡。以推进社会公平为重点的教育、文化、卫生和体育事业蓬勃发展，基本普及高中阶段教育，广泛开展大众文化活动，人人享有卫生保健服务。

　　各位代表，未来五年前景广阔、任务艰巨、使命光荣。我们坚信，经过五年的不懈努力，抚顺这座城市一定能在创造更大价值的同时，更适合人居。抚顺人一定会为工作和生活在这片美丽的土地上感到幸福和自豪!

　　2008年是全面贯彻落实党的十七大精神的重要一年，也是实施“十一五”规划的关键一年，又是新一届政府的开局之年，做好今年工作意义重大。全年经济社会发展的主要预期目标是：地区生产总值增长16%。高新技术产业增加值91亿元，增长30%。全社会固定资产投资380亿元，增长35.2%。外商直接投资8400万美元、增长22%，域外引资180亿元、增长15%。地方财政一般预算收入42.3亿元，同口径增长21.2%。全社会消费品零售总额268亿元，增长14.5%。城市居民人均可支配收入、农民人均纯收入分别增长15%和14.6%。万元生产总值能耗降低4.5%。

　　为实现上述目标，按照“好字优先、好中求快”的原则，重点做好以下工作：

　　（一）以提升质量与效益为重点，又好又快发展工业经济

　　强化项目建设。坚持扶优扶强，重点推进“千万吨炼油、百万吨乙烯”、抚铝30万吨电解铝改造二期、矿业集团10万吨小颗粒页岩炼油、红透山矿10万吨电解铜等100个大项目，促进竞争力强、带动作用大的优势骨干企业不断发展壮大。加快“两区四园”建设和招商，确保项目摆放取得突破。其中三县四区的工业园区完成项目投资105亿元，建设5000万元以上项目26个；抚顺经济开发区完成项目投资50亿元，建设超亿元项目8个。全年完成工业项目投资200亿元，增长33%。

推进科技创新。围绕石油化工、煤炭、冶金、装备制造等重点产业，特别是发挥石油化工和煤炭产学研体系完整的优势，努力突破制约资源综合利用和深度开发的技术瓶颈，加快将资源优势转化为经济优势。推进项目的引进、消化和再创新，促进科技成果向现实生产力转化，改造淘汰工艺技术落后、耗能高、污染大的设备和产品。支持引导企业争创名牌产品和驰名商标。全年开发新产品、新技术400项，实现新产品产值46亿元，增长15%。

　　加强协作配套。围绕沈阳装备、汽车制造等优势产业，充分发挥我市原材料和资源丰富的比较优势，建立完善协作配套的长期合作机制。支持地方中小企业更多进入本地大企业的采购目录和网络，鼓励大企业在同等情况下优先向地方中小企业供应原材料，形成优势互补、分工协作的双赢格局。全年实现地区产品协作配套37亿元，增长23%。

　　深化企业改革。加大重组存续国企的改制力度，尽最大努力盘活资产、保障职工利益；进一步深化建筑企业改革，稳步推进具备条件的集体企业改制；按照先易后难的原则，加快事业单位已转企的改制步伐。加强国有股权监管，确保国有股权不受侵害和国有资产保值增值。全年新增规模以上工业企业100户，规模以上工业增加值增长17%。

　　（二）以促进农民增收为重点，积极建设社会主义新农村

　　加快发展现代农业。坚持用工业理念谋划农业，围绕优势产业和特色资源，积极推进农业产业化。调整种养结构抓基地，新建标准化农业生产示范基地10个、畜禽养殖标准化小区10个，新开发林地经济30万亩，继续搞好“水果采摘月”活动。壮大企业规模抓龙头，新增规模以上龙头企业15个，实现销售收入80亿元，分别提高13.6%和14.3%。完善监管体系抓安全，加强农畜产品质量安全体系建设，提高动物疫病防控能力，确保农畜产品质量安全。健全营销网络抓市场，扶持发展农民专业合作经济组织和农村经纪人队伍，新建农民专业合作社30个。

　　加强农业基础设施建设。抓好综合开发，改造中低产田2.6万亩，生态综合治理3.6万亩；突出设施农业区域性规模发展，新建日光温室小区10个、冷棚生产小区20个；建设农村河道堤防120公里，治理小流域15万亩；新建自来水270处，解决6万人吃水难。续建南杂木至草市高速公路，开工建设永陵至桓仁段高速公路，实现村村通油路。集中力量建设新农村示范村7个、重点推进村20个、现代林业建设示范村100个。完成植树造林20万亩，封山育林30万亩。加快建设农村新能源，建成生物质气化工程10处、“一池三改”5070个。积极推进畜牧业清洁生产，重点治理规模饲养场10个。实现村村通宽带。

　　深化农村改革。坚持以工促农、以城带乡，加快生产要素有序流动和合理配置。强力推进现代林业建设，完善集体林权制度主体改革，全面深化林业配套改革，引导农民依法进行林地和土地经营权的流转，促使更多农民从事非农产业搞增收。加强村级政权组织建设，充分发挥对农民的管理、服务、引导和带领致富的作用。推进基层农业技术推广体系改革，实现一村一名技术人员。加强新型农民培训和农村劳动力转移，培训农民10万人次，转移劳动力2万人。继续实施开发式扶贫，实现稳定脱贫2.5万人。

　（三）以增强区域竞争力为重点，加快对外开放体系建设和县区经济发展

　　提高对外开放水平。继续坚持境外、域外、域内“三资”并重，瞄准国内外知名公司和龙头企业，大力引进产业带动强、科技含量高、投资强度大、资源消耗低的项目。创新利用外资方式，拓宽招商领域，突出优势产业和资源招商、特色规划招商、工业园区招商、优良环境招商，重点投向资源深加工、装备制造、现代服务、环境保护、新兴产业和现代农业，优化利用外资结构，支持现有企业增资扩股和以外引外。强化责任体系和考评激励机制，促进招商项目引得进、留得住、能发展，全年引进重点投资项目150个以上，其中超亿元项目30-50个。转变外贸增长方式，优化出口结构，完成外贸出口7.7亿美元，增长18.5%。

　　加快推进沈抚同城化。沈抚同城化对抚顺的影响和带动是全面而深刻的，其巨大作用将随着时间的推移而日渐增强，是今后工作的重中之重，必须举全市之力加以推进。今年要高标准完成沈抚连接带总体规划和分区控详规划，加快沈抚大道拓宽改造等基础设施建设，高水平打造生态景观带，启动核心试验区建设。加强与沈阳的衔接与合作，规划建设高新技术产业带，策划、包装和引进一批牵动力强的项目，形成沈抚两市产业互补、资源共享、项目集群的发展新格局。强化沈抚同城化的对外宣传，提高抚顺的知名度和影响力，吸引更多的投资者来抚投资兴业。

　　大力发展民营经济。落实政策，营造氛围，支持和推动全民创业。进一步完善扶持体系，充分发挥中小企业信用促进会和担保公司作用，解决民营企业融资难等问题，特别是对刚刚起步的民营企业给予更多扶持。引导有一定规模的民营企业走上市、参股等融资与合作之路，加强内部管理，按照现代企业制度做大做强，支持永茂建筑机械责任有限公司境外上市。民营经济实现增加值320亿元、上缴税金35亿元，分别增长20%和18%。

　　促进县区加快发展。完善相关政策，加强支持服务，进一步激发县区发展活力。支持县区依托资源优势和地域特色，因地制宜确定发展定位和方向，培育发展特色支柱产业，大力发展龙头企业和专业乡镇，形成主业突出、后续产业强劲的工业群体，引导和促进中小企业集聚发展。加快城镇化进程，加大对县城和中心镇的基础设施投入，促其完善功能、加快发展，增强承载力和辐射力。

　　（四）以旅游、商贸、房地产业为重点，促进现代服务业大发展

　　壮大旅游产业。充分发挥旅游业在现代服务业中的龙头地位，采取政府引导与市场运作相结合的投入方式，重点打造清前文化、生态娱乐、观光农业、休闲度假、红色旅游等特色旅游产品和线路，做强做大清永陵、红河峡谷漂流、皇家海洋公园、三块石森林公园和战犯管理所、平顶山惨案纪念馆等一批重点旅游项目，新增农家乐180家。加快旅游商品开发和接待服务体系建设，实施多元化旅游营销，实现旅游总收入68亿元，增长18%。

　　提升商贸流通业。结合老城区改造、新城区开发和“退二进三”，推进商业布局调整和结构优化，鼓励发展经营业态多样的商贸服务，推广连锁经营、物流配送等现代流通方式和组织形式，培育引进知名商贸企业和品牌，确保金汇广场、裕民物流等重点商业项目建成使用。大力培育新的消费热点，促使更多的本地产品就地变成商品。继续实施“万村千乡”工程，加强配送中心建设，农家店覆盖行政村达到75％。

　　发展房地产业。充分认识房地产业对推动经济、改善城市形象和人居的重要作用，坚持关心支持和规范管理并重，促进房地产业健康发展和做大做强，满足不同层次人群的住房需求。全年房地产施工面积170万平方米，竣工130万平方米。同时，加快发展法律、中介、信息、社区服务等现代服务业。

　（五）以改善城市功能和形象为重点，加强城市建设和管理

　　以一流水准规划城市。坚持以规划为龙头引领城乡建设，以新一轮城市总体规划修编为契机，高水平、大手笔编制控详规划和专项规划，建立健全城市规划管理制度，逐步形成较为完善的规划体系，重点搞好沈抚连接带、浑河两岸、高新区等规划。

　　以精品意识建设城市。积极推进城区“退二进三”，引导支持新钢铁等城区工业企业加快向外围园区搬迁，在搬迁中实现改造升级，促进城市布局调整。采取政府主导、市场运作方式，积极推进老城区危旧楼改造，达到“百姓得实惠、城市添亮点”的目的。积极推进一批重点城建项目，按照多元投资、量入为出、适度超前的原则，建设一批快捷畅通、洁净靓丽、惠民和谐项目。重点实施8大工程：以南环路东延、东环大道建设、街巷路和“摸黑路”改造等100多条道路为重点的路网建设工程；以东洲河口桥新建、搭连桥等3座桥翻建为重点的桥梁建设工程；以劳动公园二期、高尔山公园为重点的园林改造工程；以浑河南路等41条街路和完善环城林带为重点的绿化工程；以浑河中部段的水面和两岸建筑为重点的亮化工程；以排水、供水、燃气、供热为重点的200公里地下管网建设工程；以演武垃圾场建设为重点的垃圾处理工程；以28个、100万平方米住宅小区为重点的小区环境整治工程。

　　以现代理念管理城市。完善“两级政府、三级管理、四级网络”的城市管理体系，加快管理下沉，逐步建立条块结合、以块为主、运转高效的长效机制，切实杜绝城市管理的漏洞和死角。强化市容市貌综合治理，加强公用设施管理和维护，严禁乱搭乱建、乱贴乱画、乱停乱靠等行为，积极推广顺城区垃圾定时排运试点，确保主次干道人工清扫保洁率100%、生活垃圾清运率96%以上、违章建筑发现和制止率95%以上。加强社区建设，规范物业管理，创建一批整洁优美文明的“管理示范小区”。强化棚改新区硬化、绿化建设，增加公交站点。强化土地集约利用，加快土地整理和收储，努力增加土地收益，全年实现土地出让总收入突破30亿元。

　　以严厉措施治理污染。加快建设资源节约型和环境友好型社会，重点打好四个攻坚战。一是节能减排攻坚战。强化责任和激励约束机制，大力推广新技术、新产品，从源头上减少资源能源浪费和污染物排放。二是空气和水污染治理攻坚战。严格落实企业环保投入主体责任，对达不到“三同时”的新项目，坚决不予审批、不准开工生产，对污染排放超标的企业坚决停产整顿；积极实施燃煤电厂脱硫改造、东洲10万吨/日再生水厂建设、沈抚灌渠生态修复、望花地区大气污染治理等重点治污项目，确保全年化学需氧量和二氧化硫排放量分别削减2%，城区空气质量优良天数不少于310天，浑河干流城市段水质保持四类标准。三是饮用水源地保护攻坚战。严禁并坚决取缔污染水源的企业，大力推广畜禽养殖清洁生产，抓好上游南杂木镇、红透山镇、清原镇和新宾镇等4个污水处理厂建设，确保大伙房水库水质保持二类标准。四是矿山环境恢复治理攻坚战。依法规范矿山开采秩序，严禁乱采乱挖破坏植被行为，继续开展打击破坏林地专项行动；按照谁破坏、谁治理的原则，加大治理力度，逐步恢复矿山的生态环境。

　　（六）以群众得到更多实惠为重点，下更大力气改善民生

　　努力增加群众收入。以零就业家庭、棚改回迁居民为重点，加强指导、援助和培训，鼓励扶持自主创业，千方百计扩大就业再就业。实现城镇实名制就业再就业10万人以上，巩固零就业家庭至少一人就业成果，棚改回迁居民就业率达到95%以上。鼓励多种生产要素依法参与分配，拓宽城乡居民收入渠道。

　　加强社会保障体系建设。以养老保险为重点，依法强化医疗、工伤、失业等各项社会保险的扩面征收，积极推进失地农民养老保险，启动城镇居民医疗保险，实现城乡医疗保险全覆盖。加强城乡社会救助体系建设，适时提高城市低保标准，搞好低保边缘户救助。基本完成棚户区改造，建立健全廉租房和经济适用房制度，切实改善低收入家庭住房条件。新建续建住宅10.2万平方米，搬迁安置采沉区居民2365户、8700人。

　　维护社会稳定。全面落实社会治安综合治理的各项措施，巩固争创综合治理先进市成果，深化公安“三基”工程建设，进一步完善社会治安防控体系，始终保持严打高压态势，不断增强人民群众的安全感。坚持标本兼治和依法办事相结合，强化信访责任制，既拓宽社情民意表达渠道、解决上访群众合理诉求，又依法打击无理取闹违法分子、规范上访秩序。强化对全市特别是重点行业和领域的安全监管和专项整治，持续深入开展隐患排查治理，努力提高企业本质安全水平，坚决遏制较大以上事故的发生。整顿规范市场秩序，严厉打击非法集资、传销和制假售假等违法行为；严格食品药品监管，保障人民群众健康安全。完善公共突发事件预警系统和各类应急预案，提高保障公共安全和处置突发事件的能力。深化“五五”普法，加强法律服务和法律援助。

　　（七）以促进社会公平为重点，全面发展各项社会事业

　　优先发展教育事业。加大公共财政投入，优化中小学布局，扩大优质教育资源，加强薄弱学校改造建设，提高教师队伍素质，符合条件的城区教师交流比例达到20%，促进教育公平。推进农村义务教育经费保障机制改革，做好城市义务教育学生免杂费工作。调整优化职业院校专业布局，全面推进“校企合作”和“工学结合”，职业院校毕业生就业率达到85%以上。支持辽宁石油化工大学等高等院校加快发展。牢固树立人才是第一资源的观念，完善人才评价、选拔任用和激励机制，用好现有人才，培养适用人才，引进紧缺人才，营造人尽其才、才尽其用的良好环境。

　　大力发展文化卫生体育事业。弘扬雷锋精神，提高市民素质，加强引导、教育和整治，努力消除破坏公物、不讲公德等不文明行为。积极开展形式多样、健康向上的群众性文化活动，鼓励创作优秀剧目和文艺精品。以解决群众看病难、看病贵为重点，在城市加强公共卫生体系和医疗服务体系建设，逐步实现小病在社区、大病进医院；在农村扩大新型合作医疗覆盖面，提高参合率。新建全民健身场所80处，承办好全国第15届冬泳锦标赛和第六届全国少儿乒乓球杯总决赛等赛事。整合广电资源，提高节目质量。稳定低生育水平，提高出生人口素质，人口出生率控制在5.94‰以内。进一步做好民族宗教、国家安全和双拥共建等各项工作。

　　（八）以服务经济社会为重点，进一步做好财税金融工作

　　促进财税收入平稳较快增长。巩固完善市与县区财税新体制，进一步营造千方百计快发展、培财源的良好氛围，做大地区财源规模。全面落实促进经济增长的各项财税政策，发挥财政资金的杠杆作用，促进企业发展和税收增长。加强税源管理和纳税评估，堵塞征管漏洞，做到应收尽收和均衡入库。启动财税银横向联网，推进县区国库集中支付制度和乡财县管改革。

　　优化财政支出结构。扩大部门预算编制范围，积极推进预算单位银行公务卡和政府集团采购，特别是加强通讯、车辆、公务接待等方面的管理，努力降低行政成本。从严控制一般性支出，扩大改善民生支出。增强预算的规范性和透明度，提高财政资金的使用效益。

　　加强与金融机构合作。全方位扩大与开发银行合作，促进地方商业银行加快发展；加强与驻抚金融机构的协调沟通，促其加大对地方发展的支持。优化金融环境，打造诚信社会，做到适度举债、科学还债。鼓励支持保险业健康发展，不断满足社会需求。

　（九）以提高行政效能为重点，强化政府自身建设

　　依法行政，增强政府决策力。贯彻落实《全面推进依法行政实施纲要》，严格按照法定的权限和程序行使权力、履行职责。健全重大事项集体决策、专家咨询、社会公示与听证、决策评估等制度，提高立法质量和决策水平。自觉接受人大及其常委会的法律监督和政协的民主监督，认真听取民主党派、工商联、无党派人士和各人民团体的意见，努力使政府决策顺民心、合民意，让各项工作经得起实践、人民和历史的检验。

　　开拓进取，增强政府创新力。坚持把创新贯穿于政府工作的各个环节，不断解放思想，以振兴抚顺为己任，以强烈的主人翁意识和责任感、危机感认真履行职责，以更加宽广的视野和战略思维谋划推进工作，坚决克服封闭保守、墨守成规等思想障碍，勇于改革创新，勇于冲破“条条”和“框框”的束缚，切实增强抓项目的本领、解难题的能力、促发展的水平，努力实现又好又快发展。

　　提质提速，增强政府执行力。政府各项工作都要高标准、严要求、快节奏，真抓实干，少说多做，务求实效。继续深化行政审批制度改革，简化办事程序，优化工作流程，推进政务公开，提高工作效率和服务质量，更好地为市民、企业和社会服务。坚持把抓落实摆在政府工作首位，以积极负责的态度、只争朝夕的精神，把各项任务落实到每个月、每一天，落实到每个单位、每个人。强化团结协作，强化督查督办，强化考核奖惩，强化行政问责，激励多干事、褒奖干成事、治理不作为、惩戒乱作为，扎扎实实、保质保量地干好每一项工作。

　　勤政廉政，增强政府公信力。牢固树立人民政府为人民的理念，坚持以改善民生、促进民和、确保民安为己任，任何时候、任何工作都要以人为本、心系群众、服务百姓，促进社会公平公正。加快建设诚信政府，凡是在人代会上向全市人民做出的报告，凡是政府常务会议和市长办公会议决定的事项，凡是对企业、对群众做出的承诺，都必须不折不扣地落到实处。全面落实廉政建设责任制，加强对权力运行的监督制约，让权力在阳光下运行。坚决纠正损害群众利益的不正之风，对工程建设、土地出让等重点领域加大监管力度，对违法违纪案件和人员坚决查处，绝不姑息。

　　各位代表，抚顺正处于加快发展的关键时期，抚顺已踏上奋力赶超的历史征程。我们要在市委的正确领导下，在市人大、市政协的监督支持下，全面贯彻落实科学发展观，倍加珍惜来之不易的好形势，倍加珍惜团结和谐的好局面，把人民的期望当作前进的动力，把崇高的使命化为积极的行动，鼓实干之劲，造发展之势，聚和谐之力，谱写发展的新篇章，创造振兴的新辉煌！
